# Coagulation Profile of Convalescent Plasma Donors and Recipients

**DOI:** 10.1177/10760296251317522

**Published:** 2025-01-31

**Authors:** Hanna H Pitkänen, Tuukka Helin, Tamim Khawaja, Jukka-Pekka Pietilä, Mikael Kajova, Hanna Välimaa, Tero Vahlberg, Jarkko Ihalainen, Antti Vierikko, Olli Vapalahti, Anu Kantele, Riitta Lassila

**Affiliations:** 1 Helsinki University Hospital, Division of Anesthesiology, Department of Anesthesiology, Intensive Care and Pain Medicine, University of Helsinki and Helsinki University Hospital, Helsinki, Finland; 2 Department of Hematology, Coagulation Disorders Unit, Helsinki University Hospital, Helsinki, Finland; 3 Research Program in Systems Oncology, Faculty of Medicine, 3835 University of Helsinki, Helsinki, Finland; 4 Department of Clinical Chemistry, HUS Diagnostic Centre, Helsinki University Hospital, and University of Helsinki, Helsinki, Finland; 5 Meilahti Vaccine Research Center, MeVac, 159841 University of Helsinki and Helsinki University Hospital, Helsinki, Finland; 6 Department of Infectious Diseases, 159841 University of Helsinki and Helsinki University Hospital, Helsinki, Finland; 7 Human Microbiome Research Program, Faculty of Medicine, 3835 University of Helsinki, Helsinki, Finland; 8 FIMAR, Multidisciplinary Center of Excellence in Antimicrobial Resistance Research, 3835 University of Helsinki, Helsinki, Finland; 9 Department of Biostatistics, University of Turku and Turku University Hospital, Turku, Finland; 10 99296 Finnish Red Cross Blood Service, Vantaa, Finland; 11 11326 Finnish Medicines Agency, Helsinki, Finland; 12 Viral Zoonoses Research Unit, Departments of Virology and Veterinary Biosciences, University of Helsinki and Helsinki University Hospital Diagnostic Center, Helsinki, Finland

**Keywords:** convalescent plasma, COVID-19, von Willebrand factor, thrombin, thromboprofylaxis

## Abstract

Convalescent plasma (CP) therapy for COVID-19 infection may have favorable safety but varying efficacy, with concerns about its procoagulant impact. We investigated whether administration of CP to hospitalized patients affects their coagulation profile. Fifty-four patients randomized in a double-blinded fashion received either placebo, low-titer CP (LCP) or high-titer CP (HCP). Donor blood samples were obtained at the time of the plasmapheresis, while recipient blood samples were collected before infusion, one day post-infusion and between two and six days after infusion. Routine laboratory follow-up, coagulation biomarkers, antiphospholipid antibodies, and thrombin generation (TG) were assessed. CP donors had normal blood cell counts and coagulation profiles, without differences between LCP and HCP donors at the baseline. All CP recipients were on low-molecular-weight heparin thromboprophylaxis at the time of the infusion. Despite randomization, the HCP group had lower baseline (p = 0.004) and Day 1 platelet counts (p = 0.019) than the LCP group. Von Willebrand antigen (VWF:Ag) levels clearly exceeded normal without differences at baseline. At Day 1, LCP recipients had higher VWF:Ag (mean ± SD 224 ± 15%) than HCP recipients (210 ± 8%) (p = 0.012). In all groups, overall 80% lupus anticoagulant was positive. Baseline TG variables were comparable, but again LCP recipients exhibited higher endogenous thrombin potential (ETP) (1313 ± 535 nM.min) (p = 0.038) and peak TG (184 ± 106 nM) (p = 0.037) than the HCP group (870 ± 425 nM.min and 86 ± 54 nM). Our findings show that LCP increases VWF:Ag levels and enhances TG despite the thromboprophylaxis. These results suggest that HCP induces less hypercoagulability than LCP, which may contribute to the variability in CP efficacy.

## Introduction

The coronavirus disease 2019 (COVID-19) pandemic reignited an interest in passive immunization using convalescent plasma (CP) therapy, a therapeutic approach with a history of over a century.^1^ In August 2020, the US Food and Drug Administration (FDA) authorized emergency use of CP for patients with severe or life-threatening COVID-19.^2^ Although COVID-19 CP has demonstrated a favorable safety profile, its efficacy has been inconsistent and concerns remain about its potential procoagulant impact, particularly among patients with a severe disease, which predisposes to thrombotic complications.^3–5^ The effectiveness of CP in treating COVID-19 has been investigated in numerous randomized trials.^6^ CP has been shown to reduce the risk of hospitalization when given to outpatients early in the course of the disease.^7,8^ However, for hospitalized patients with severe and progressed disease and high autologous antibody titers, CP treatment does not appear to offer clinical benefits as reported by several studies,^9–14^ although one large study reported decreased mortality rates in mechanically ventilated patients with ARDS after high-titer CP (HCP) infusion.^15^ Current guidelines recommend that only HCP should be used, since negative results often included patients who received low-titer CP (LCP) and HCP is comparatively resistant to viral escape.^3,16^

The primary objective of this study was to observe the possible effects of convalescent plasma on the coagulation system during the acute phase of COVID-19 infection. During early pandemic, we investigated how administering CP to hospitalized patients within 10 days of symptom onset impacted their coagulation profile. Fifty-four patients were randomized to receive placebo, LCP or HCP. As the source of plasma, we also evaluated the coagulation profiles of all 21 CP donors. To our knowledge, this is the first study to examine the interactions between CP and the recipient's blood coagulation activity and comparing the effects of placebo (0.9% saline), LCP, and HCP.

## Patients, Materials and Methods

### Trial Design

This study was conducted as a sub-study of a prospective double-blinded randomized controlled study on the efficacy and safety of CP treatment of hospitalized adult COVID-19 patients at the Helsinki University Hospital (later HUS) between February 2, 2021, and January 19, 2022. The detailed description of the trial design has been previously published.^17^ Blinded study personnel screened daily the electronic patient records of RT-PCR -confirmed COVID-19 patients who were hospitalized on regular wards in HUS hospitals. Possibly eligible patients according to inclusion and exclusion were interviewed by a blinded study nurse or a researched and asked the patient to provide a written informed consent if confirmed eligible. The recipients were randomized 1:1:1 to receive either placebo (0.9% saline), LCP or HCP by unblinded study personnel using Granitics CFR software with a fixed block size of two times the number of subject groups. Eligibility was reconfirmed immediately prior to infusion of the study product. Unblinded study nurses administered the infusion on the day of recruitment or at latest the following day by. Apart from randomization and infusion, the unblinded study personnel did not take part in the study conduct or patient care. Blinded physicians and nurses handled the recruitment and follow-up, and standard of care was provided by hospital staff.

The titers of CP were categorized by the levels of neutralizing antibodies (NAb) against the SARS-CoV-2 wild-type virus (Wuhan-like original variant) as measured by a microneutralization test (MNT) from the donated CP. Plasma with a NAb titer of 1:20–1:80 against the wild-type virus by MNT was LCP, and that with ≥1:160 HCP.

The 21 donors were recruited among convalescent COVID-19 patient in the Helsinki metropolitan area who had tested positive for SARS-CoV-2 by reverse transcription PCR (RT-PCR) from a nasopharyngeal swab sample. The final participant group of the main study comprised 57 patients of whom 54 were eligible for the coagulation based sub-study. Recipients were recruited from ≥18-year-old patients hospitalized for RT-PCR-confirmed COVID-19 at HUS. For inclusion, symptom duration had to be less than 10 days. The exclusion criteria included current systemic corticosteroids, immunosuppression or immunosuppressive/-modulating therapy over the past six months and do-not-resuscitate order or a decision to withhold ICU treatment.^17^

The ABO and Rh blood groups were determined from all participants on enrollment using routine red blood cell agglutination technique (Bio-Rad, Basel, Switzerland). Unblinded study personnel randomized the patients after verifying the availability of both LCP and HCP blood type-compatible plasmas. Placebo was administered to 19 recipients, 18 received LCP and 17 HCP. The recipients received a single intravenous 60-min infusion of 200 mL CP or saline, administered by unblinded study nurses. The degree of respiratory failure was recorded according to the WHO clinical progression scale score.^18^

In addition to analyzing the study groups based on the CP product or placebo they received, we also studied the patients according to 1) the thromboprophylaxis of LMWH with a standard or intermediate dose, and 2) whether oral dexamethasone was administered or not.

### Ethics Approval and Monitoring

Our study was approved by the ethics committee of Helsinki University Hospital (HUS/1637/2020), and written informed consent was obtained from all participants in accordance with the Declaration of Helsinki. The study was monitored by an external monitor (HUCH Institute Ltd), and it was registered at Clinical Trials (NCT04730401).

### Preparation of Plasma Products at the Finnish Red Cross Blood Service (FRCBS)

The prospective donors were prescreened by the clinical research group as described earlier.^17^ The donors arrived to the FRCBS apheresis unit for a pre-apheresis visit, where their eligibility to donate plasma was confirmed and samples were collected for routine blood donor screening according to the FRCBS guidelines.^19^ The presence of HLA antibodies from women, and men who had previously received blood products was excluded. The recommendations of the European Commission on CP collection and the guidance of the EDQM were followed.^20–22^

The plasmapheresis was performed with TerumoBCT Trima 6.0 or 7.0 machines (TerumoBCT, Lakewood, USA) with standard plasmapheresis settings using a Terumo Multiplasma collection kit (Cat nro 82700). Plasma was aliquoted into three 200 ml bags in a closed process, the units were labeled and frozen to −25 °C within 6 h from plasmapheresis. The units were stored and distributed from FRCBS frozen to the HUS blood bank where they were thawed for transfusion as a part of hospital routine.

### Blood Sample Collection

The donor blood samples were obtained at the time of the plasmapheresis.

Recipient blood samples were collected as follows: before infusion (Day 0, baseline), one day after infusion (Day 1), and from two to six days after infusion (Day 2–6). For coagulation studies, blood was collected in sodium-citrate anticoagulant (3.2% Na-citrate). Platelet-poor plasma (PPP) was processed by centrifugation at 2500 g for 15 min at room temperature and separated plasma samples were stored in aliquots at −80 °C.

### Routine Laboratory Follow-up, Coagulation Biomarkers, Antiphospholipid Antibodies, and Thrombin Generation

Routine laboratory tests were done in the Helsinki University Hospital laboratory, using commercial reagents with locally verified reference intervals. Coagulation tests prothrombin time (PT, % from normal plasma), activated partial thromboplastin time (APTT, s), thrombin time (TT, s), antithrombin, D-dimer, coagulation activity of factor VIII (FVIII), fibrinogen and von Willebrand factor antigen (VWF:Ag, %) were measured with ACL TOP 750^®^ analyzer, in all using Instrumentation Laboratory^®^ reagents (Werfen, Barcelona, Spain). Lupus anticoagulant testing was done with Siemens BCS XP^®^ analyzer, using both Russel-Viper-Venom time (RVVT) and APTT -based assays, with both screening and confirmation tests, consistent with the ISTH guideline for lupus anticoagulant (LAC) (Siemens Healthineers, Erlangen, Germany).^23^ Cardiolipin and beta-2-glycoprotein IgG antibodies were analyzed with Phadia 250^®^ immunoanalyzer (Thermo Fischer, Waltham, MA, USA). Blood cell counts were measured with Sysmex XN-1000^®^ analyzer (Roche, Basel, Switzeland) and C-reactive protein (CRP) was assessed with Siemens Atellica^®^ analyzer (Siemens Healthineers, Erlangen, Germany).

Thrombin generation in plasma was triggered with 1 pM TF, without thrombomodulin addition and was measured with the Calibrated Automated Thrombogram (CAT) assay, as previously reported.^24^ The CAT variables included lag time (time to initiation of thrombin formation, min), endogenous thrombin potential (ETP; the area under the curve; nM.min), peak thrombin (maximum thrombin concentration, nM) and time to peak thrombin (ttpeak, min). As reference for CAT, control plasma was collected from 12 non-matched healthy volunteer donors.

### Statistics

Most of the continuous variables were non-normally distributed, and non-parametric tests were used for all continuous variables. The differences in continuous variables between the groups were tested using Kruskal-Wallis test with Dunn-Bonferroni correction applied for pairwise comparisons. The Pearson Chi-Square test or Fisheŕs exact was used to analyze categorical variables. P-values below 0.05 were considered statistically significant, and two-sided tests were employed. Statistical analyses were carried out with IBM SPSS Statistics 29 (IBM Corp., Armonk, NY).

### Power Calculations

The participant group size of 130 was calculated for the original study as follows.^17^ Minimal clinically important difference in starting systemic corticosteroid for aggravation of COVID-19 was set at 45% relative reduction (RR = 0.55) and was expected for 35% of patients in the placebo group and 19% of patients in the HCP group. A total sample size of 372 (124 per group) was needed when using 80% power and significance level of 0.05; six dropouts per group were allowed, thus arriving at the 130 participants per group or 390 participants in total. We, however, decided to target at a round number of 400 participants.

## Results

### Donors

Demographic and clinical data of the donors of CP are presented in Table 1. Only four donors were female. Most donors (19/21) had mild disease with WHO clinical progression score of 4–5. Two donors were intubated (score 8) (Table 1). Coagulation biomarkers did not differ between LCP and HCP donor plasmas, and median coagulation biomarkers were within the reference interval, when applicable. All donor plasmas were lupus anticoagulant negative. CAT variables did not differ between LCP and HCP donors (Table 2).

**Table 1.** Donor Demographic and Clinical Data.

Variable	n = 21
**Age (years)**	40 [30–52]
**Female**	4 (19)
**BMI (kg/m^2^)**	31 [27–37]
**Comorbidity**	
Cardiovascular	4 (19)
Respiratory	3 (14)
**Antibiotics for respiratory infection**	8 (38)
**Medication not for COVID-19**	6 (29)
**Non-hospitalized**	10 (48)
**Maximal respiratory support requirements**	
None	12 (57)
Nasal cannula/mask ≤ 5 l O_2_	4 (19)
Nasal cannula/mask > 5 l O_2_	3 (14)
Intubation	2 (10)
**Highest WHO clinical progression scale score**	
Hospitalized; no oxygen therapy (4)	12 (57)
Hospitalized; oxygen by mask or nasal progs (5)	7 ((33)
Mechanical ventilation pO_2_/FIO_2 _< 150 mmHg or vasopressors (8)	2 (10)
**Anticoagulation during acute phase**	
No	12 (57)
Standard prophylactic LMWH (0.5 mg/kg QD)	2 (10)
Intermediate prophylactic LMWH (0.5 mg/kg BID)	7 (33)

The results are presented as median [interquartile range] for continuous variables, and number (percentage) for categorical variables. BMI, body mass index; LMWH, low-molecular-weight heparin; QD, once daily; BID, twice daily

**Table 2.** Donor Median (Minimum-Maximum) Coagulation Variables.

Biomarker	Normal range	LCP	HCP
PT (%)	70–130	95 (76–129)	86 (72–129)
APTT (s)	23–33	28 (26–33)	30 (25–35)
TT (s)	17–25	22 (19–26)	23 (19–49)
AT (%)	84–108	98 (80–108)	93 (81–103)
Fibrinogen (g/L)	2–4	2.4 (1.8–3.8)	2.4 (1.7–4.6)
FVIII (%)	70–160	153 (92–224)	130 (82–180)
VWF:Ag (%)	50–190	131 (81–194)	129 (65–183)
D-dimer (mg/L)	<0.5	0.2 (0.2–0.5)	0.3 (0.1–0.9)
CAT lagtime (min)		5.5 (4.4–9.7)	5.4 (5.0–5.7)
CAT ETP (nmol/min)		801 (495–1804)	815 (482–1001)
CAT peak (nmol/L)		75 (39–191)	74 (37–98)
CAT time to peak (min)		11.0 (9.6–15.6)	12.0 (10.2–12.7)

LCP: low-titer convalescent plasma group, HCP high-titer convalescent plasma group. PT: prothrombin time (% from normal plasma), APTT: activated partial thromboplastin time, TT: thrombin time, AT: antithrombin, FVIII: coagulation activity of factor VIII, VWF: von Willebrand factor antigen. CAT: Calibrated Automated Thrombogram, ETP: endogenous thrombin potential.

## Study Groups

### Demographics

Recipient demographic and clinical data are shown in Table 3. Most recipients (46/54) had mild disease with WHO clinical progression scale score 4–5. Four required high flow support or non-invasive ventilation (score 6). Three patients were intubated (score 8), and one patient from LCP group died by Day 30 (score 10). All recipients were anticoagulated with subcutaneous LMWH thromboprophylaxis, with either standard (0.5 mg/kg once daily) thromboprophylaxis or an intermediate dose (0.5 mg/kg twice daily) (Table 3). According to our hospital policy, enoxaparin was used as LMWH product. Anti-Factor Xa was not measured, while only standard and intermediate doses were administered and no severe renal impairment occurred. Three recipients had oral platelet antagonist at the time of hospital admission, but adverse outcomes during the 30-day follow-up were not observed. The recipients were not screened for asymptomatic venous thromboembolism (VTE), and criteria for imaging studies included clinical suspicion of VTE only. Thrombosis was not encountered during the 30-day follow-up period. The need for dexamethasone therapy and LMWH dose were based on clinical assessment. Comorbidities, LMWH dosing and need for dexamethasone therapy were all equally distributed between the study groups. Median duration of hospitalization was relatively short (4 days), which resulted in considerable loss of follow-up blood samples after Day 1, since blood samples were collected only during hospital stay.

**Table 3.** Recipient Demographic and Clinical Data.

Variable	n = 54
**Age (y)**	50 [39–65]
**Female**	24 (44)
**BMI (kg/m^2^)**	28 [25–33]
**ABO blood group**	
O	15 (28)
A	20 (37)
B	16 (30)
AB	3 (5)
**Comorbidity**	
Cardiovascular	19 (35)
Diabetes	7 (13)
Renal dysfunction	2 (4)
Respiratory	12 (22)
Obesity	23 (43)
**Antibiotics for respiratory infection**	26 (48)
**Medication not for COVID-19**	26 (48)
**Received vaccination before infusion**	10 (19)
**Respiratory requirements at infusion time**	
No Oxygen	30 (56)
Oxygen by mask or nasal prongs	24 (44)
**Highest WHO clinical progression scale score by day 30**	
Hospitalized; no oxygen therapy (4)	21 (39)
Hospitalized; oxygen by mask or nasal progs (5)	25 (46)
Hospitalized; oxygen by NIV or high flow (6)	4 (7)
Mechanical ventilation pO_2_/FIO_2 _< 150 mmHg or vasopressors (8)	3 (6)
Dead (10)	1 (2)
**Routine laboratory values at infusion time**	
CRP (mg/L)	51 [17–79]
Hemoglobin (F: 117–122 g/L, M: 134–167 g/L)	136 [127–144]
WBC count (3.4–8.2×10^9^/L)	4.2 [3.4–5.5]
Platelets (150–360×10^9^/L)	163 [125–202]
Creatinine (F: 50–90 µmol/L, M: 60–100 µmol/L)	62 [56–78]
**Anticoagulation at infusion time**	
Standard prophylactic LMWH (0.5 mg/kg QD)	35 (65)
Intermediate prophylactic LMWH (0.5 mg/kg BID)	19 (35)
**Oral platelet inhibitor**	3 (6)
**Intubation at day 30**	3 (6)
**Death at day 30**	1 (2)
**Duration of hospital stay (d)**	4 [3–7]
**Thrombosis by day 30**	0
**Dexamethasone**	18 (33)
Day dexamethasone was given after infusion	1 [1–2]

The results are presented as median [interquartile range] for continuous variables, and number (percentage) for categorical variables. BMI, body mass index; NIV, non-invasive ventilation; CRP, C-reactive protein; WBC, white blood cell count; LMWH, low-molecular weight heparin; QD, once daily; BID, twice daily. Obesity is defined by BMI > 30 kg/m^2^

### Blood Cell Count, Coagulation Biomarkers and Antiphospholipid Antibodies

**Figure 1.**
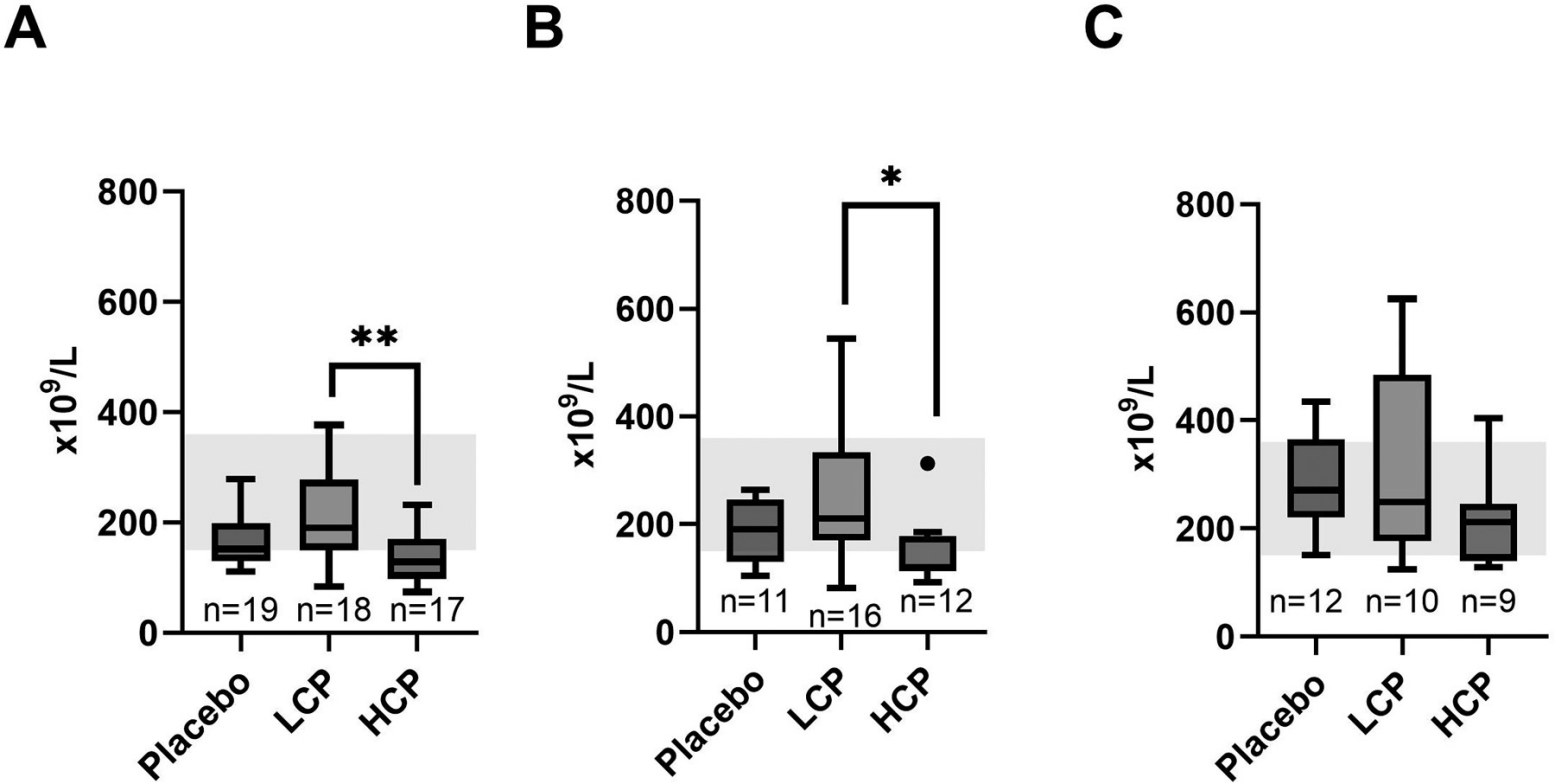
Recipient platelet levels in baseline (A), Day 1 (B) and Day 2–6 (C). Baseline platelet count (A) was higher in the low-titer convalescent plasma (LCP) group than in the high-titer convalescent plasma (HCP) group, and the difference, although less, was still present on Day 1 (B). Reference range is illustrated in grey background. n = the number of recipients in each study group.**p = 0.004 *p = 0.019.

C-reactive protein (CRP), hemoglobin, and white blood cell count did not differ between the study groups. Platelet count was within reference range in LCP and placebo groups, but HCP group presented with mild thrombocytopenia (mean ± SD: 132 ± 43×10^9^/L, reference range 150–360 × 10^9^/L) at baseline and on Day 1 (148 ± 210×10^9^/L) (Figure 1 A-B). Baseline platelet count was higher in LCP group compared with HCP group (p = 0.004) (Figure 1A). The difference, although less significant, persisted on Day 1 (p = 0.019) (Figure 1B). On Day 2–6 there was no difference in platelet counts between LCP and HCP groups (Figure 1C). Platelet counts between placebo group and HCP or LCP, however, did not differ (Figure 1A-C).

**Figure 2.**
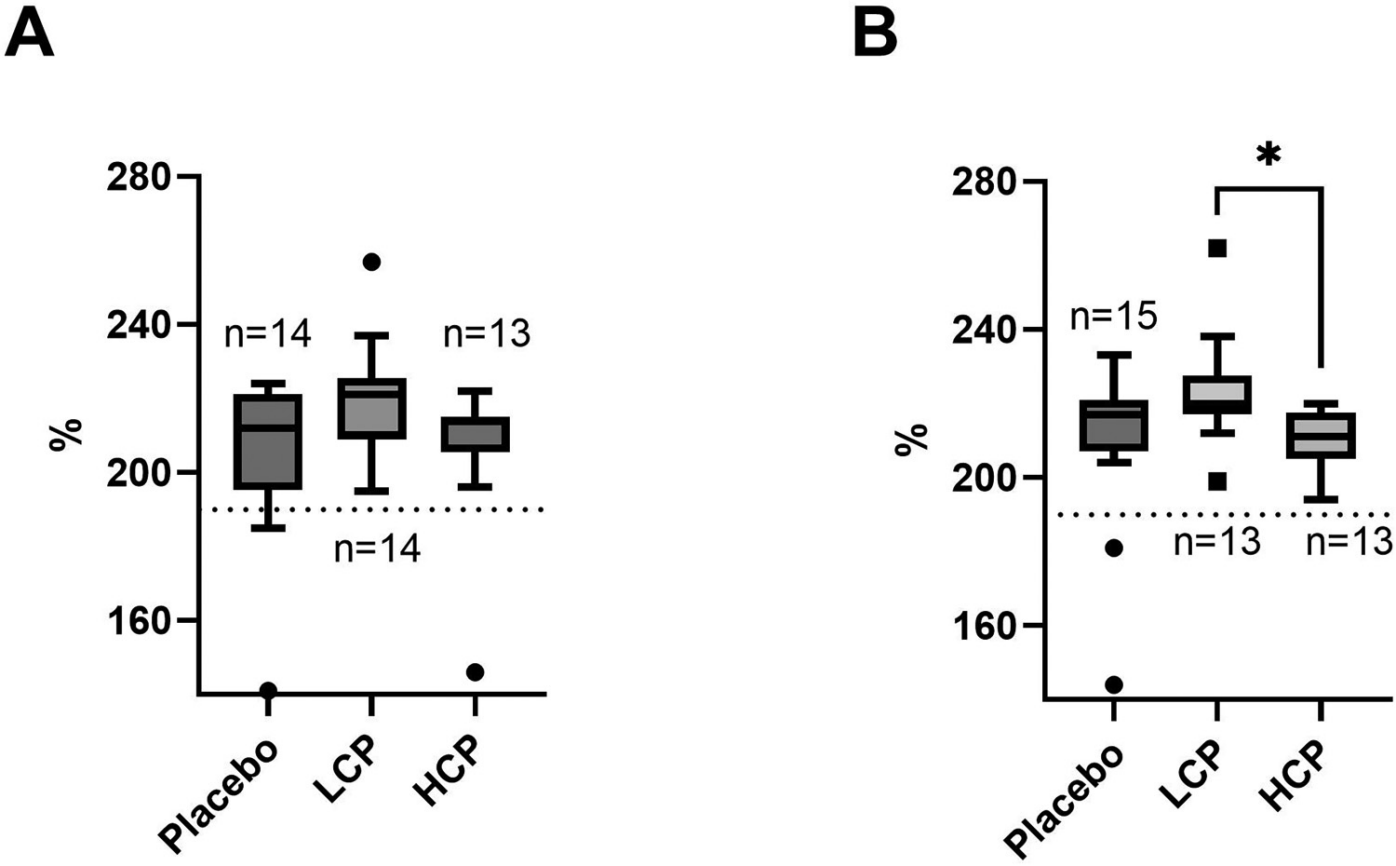
Recipient baseline (A) and Day 1 (B) von Willebrand Factor antigen (VWF:Ag) levels. There was no baseline VWF:Ag level differrence between the study groups (A). On Day 1 VWF:Ag was higher in the low-titer convalescent plasma (LCP group) than the high-titer convalescent plasma (HCP) group (B). The stratified line illustrates the upper limit of the reference range for VWF:Ag. n = the number of recipients in each study group. *p = 0.012.

The VWF:Ag levels were quite similar between the groups at baseline (Figure 2A). However, on Day 1 VWF:Ag was higher in the LCP group than in the HCP group (p = 0.012), while no difference was observed between the LCP and placebo, or HCP and placebo groups (Figure 2B). Importantly, in all study groups, the median VWF:Ag exceeded the reference range both at baseline and on Day 1 (Figure 2A-B). Although, there was an overall group difference in FVIII (p = 0.04) on Day 1, after Bonferroni correction FVIII levels in LCP group were not significantly higher compared to HCP (p = 0.06), and no differences between FVIII levels in LCP and placebo or HCP and placebo groups were observed. Unlike VWF:Ag, FVIII activity was within the reference interval (data not shown). PT, APTT, TT, AT, fibrinogen, or D-dimer did not differ between the groups, and the values remained in the reference interval.

Most of the recipients (81%) were LAC positive at the baseline. Recipient LMWH anticoagulation likely influences these findings. The percentage of LAC positive patients prevailed in the study groups on Day 1 without differences between the groups. Anti-β2-glycoprotein and anti-cardiolipin antibodies remained negative in all study groups.

### Thrombin Generation

**Figure 3.**
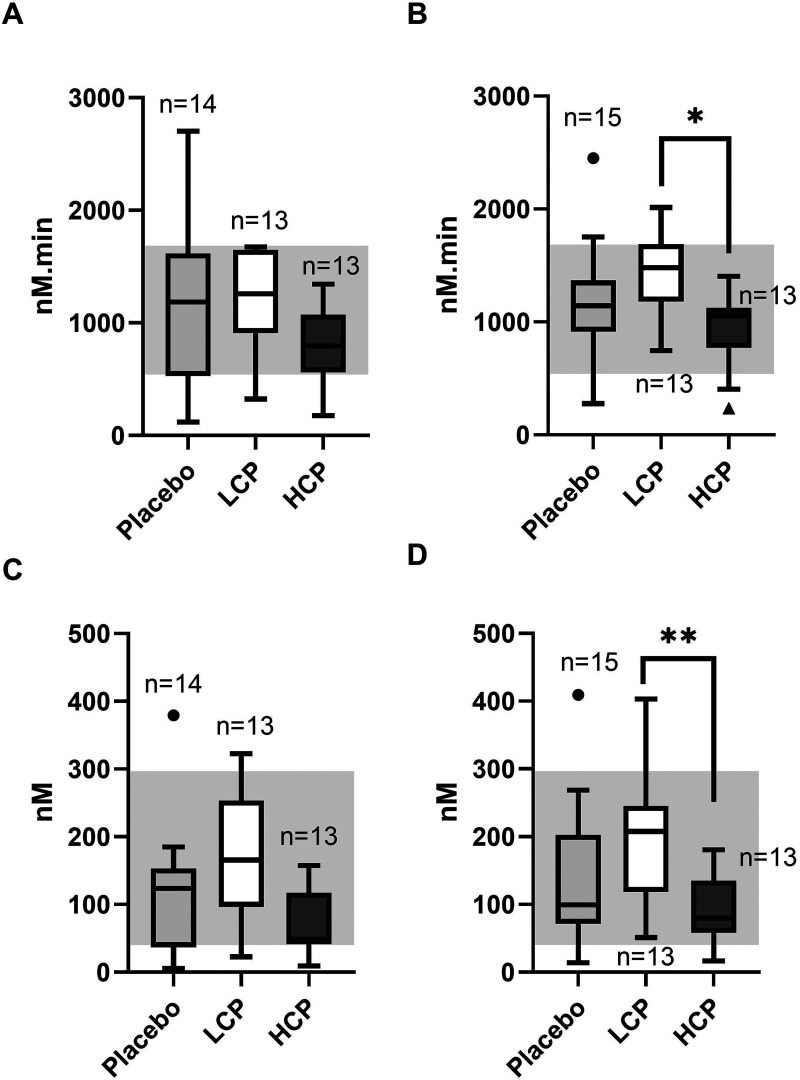
Recipient baseline and Day 1 endogenous thrombin generation (ETP) (A-B) and peak thrombin generation (C-D) measured with Calibrated Automated Thrombogram. There was no difference between the groups in baseline ETP (A). On Day 1 (B), ETP showed enhanced thrombin generation in the recipients of the low-titer (LCP) compared to the high-titer convalescent plasma (HCP). Peak thrombin generation levels were similar between the groups at baseline (C), but again at Day 1 peak thrombin was higher in the LCP than the HCP group (D). Thrombin generation variable ranges (min-max) from 12 healthy controls are illustrated in grey. n = the number of recipients in each study group.*p = 0.038 **p = 0.037.

Baseline thrombin generation (TG) variables were aligned between the groups (Figure 3A and 3C). However, both ETP and peak thrombin generation indicated more TG on Day 1 in the LCP group than in the HCP group (p = 0.038 and p = 0.037, respectively) (Figure 3B and 3D). Overall, we did not observe differences in TG between placebo group and HCP and or LCP groups (Figure 3A-D).

### LMWH and Dexamethasone

The proportion of the two dosing regimens were similar within the HCP/LCP/placebo groups. Overall, the majority of the recipients (35/54) received a standard dose LMWH (0.5 mg/kg once daily), while 19/54 of the recipients received an intermediate dose (0.5 mg/kg twice daily) due to their high-risk profile for VTE (Table 3). On Day 1, those in the intermediate LMWH dose group had higher CRP levels (p = 0.026) (Supplemental Figure 1A), and their CAT lag time was prolonged (p = 0.002) (Supplemental Figure 1C) compared with the standard LMWH dose group. Furthermore, the baseline peak thrombin generation was lower (p = 0.032) (Supplemental Figure 1B), and time to thrombin peak (ttPeak) was longer in the intermediate than the standard group (p = 0.004) (Supplemental Figure 1D). Other coagulation biomarkers were not influenced by the two LMWH regimens.

Dexamethasone was administered to 18/54 recipients (Table 3). There were no proportional differences in dexamethasone administration between the CP groups. Fifteen patients received dexamethasone one to two days after infusion, two received it three days after infusion, and one patient after seven days. Blood samples on Day 1 were collected from all patients before dexamethasone was administered. Of the 18 recipients receiving dexamethasone, seven were on standard dose LMWH, while the remining 11 were on the intermediate dose, compatible with the inflammatory risk profiles of the patients.

## Discussion

All CP donors had normal blood cell counts and coagulation profiles, without differences in coagulation activity between LCP and HCP donors at the baseline. In contrast, baseline VWF:Ag was elevated across all CP recipients with acute COVID-19 infection, as has been previously reported.^25^ However, LCP further increased VWF:Ag levels and also profiled as having enhanced thrombin generation in response to tissue factor in CAT assay. Despite randomization, the HCP group had lower baseline and Day 1 platelet counts than the LCP group, though without thrombocytopenia, and no differences between placebo group versus LCP or HCP groups were noted. Most recipients were at baseline and after CP infusion LAC positive, which LMWH anticoagulation likely influences as it may induce false positive LAC.^26^ Overall, our findings suggest that HCP administration has a more favorable profile than LCP, aligning with the guidelines recommending high-titer anti-SARS-CoV2 for treatment.^16,27^

The axis of platelets, VWF and VWF multimer proteolysis by ADAMTS13 has attracted special interest in COVID-19, as elevated VWF:Ag levels have been associated with more severe disease in COVID-19, likely due to sustained endothelial perturbation, and cellular activation.^28,29^ In case of acute coronary syndrome, but not in COVID-19-associated coagulopathy, enoxaparin has been shown to limit the early rise in VWF:Ag levels.^30^ In our study, LCP triggered both increased VWF:Ag levels and enhanced thrombin generation, potentially creating a hypercoagulable state despite LMWH therapy, whereas HCP and placebo did not. This suggests that LCP administration could be even harmful for COVID-19 patients at higher risk of microthrombosis and thromboembolic events, although we did not detect thrombosis among the CP recipients. Thrombocytopenia is a known clinical manifestation of severe or progressive COVID-19 and other severe thrombo-inflammatory conditions.^31,32^ Although there was a baseline difference between the two groups despite randomization, the rising platelet counts in HCP group may reflect a beneficial effect of CP. While transient antiphospholipid antibody positivity has been observed in the acute phase of COVID-19, a causal relationship between this and thrombotic complications has yet to be confirmed.^33^ Most recipients in our study were LAC-positive at baseline and remained so after CP or placebo infusion, indicating that CP does not immediately alter phospholipid antibody status. LAC-positivity is likely explained by the combination of acute phase of COVID-19 and LMWH anticoagulation.^26,33,34^ To add, all CP donors were LAC-negative at the time of plasmapheresis.

All recipients were anticoagulated with LMWH at the time of the CP or placebo infusion according to the recommendations which were later available.^35^ The recipients with higher CRP counts were on intermediate LMWH dosing scheme and received dexamethasone, which led to the anticipated reduction in thrombin generation. Thromboembolic complications were not observed during the 30-day follow-up period, aligning with an efficacious thromboprophylaxis. Coagulation biomarkers, particularly VWF:Ag or FVIII neither showed significant differences between those who received dexamethasone and those who did not. This observation suggests that the combination of anti-inflammatory medication and LMWH may help to stabilize endothelial functions and mitigate hypercoagulability. Acute COVID-19 infection increases the risk of thromboembolic events.^36^ In our study all patients received LMWH prophylaxis, and dexamethasone was administered if clinical decline was developing. LCP induced less favorable coagulation profile than HCP, characterized by elevated levels of VWF:Ag and enhanced thrombin generation. Despite the differences between the two CP products, we did not observe symptomatic thrombotic complications in any of the study groups. This could be due to the thromboprophylaxis, but it has been previously shown that individuals with an asymptomatic or mild disease who are not hospitalized have low rate of thromboembolic complications, while patients who required treatment in intensive care unit have 2.5 times increase in the incidence of VTE.^37–39^ Our study population was relatively stable, 85% had WHO clinical progression score scale of 4 to 5. Only one recipient died and three were intubated. This suggests that our study cohort had a relatively small risk for thromboembolic complications. Overall, our observations relate to laboratory findings and do not indicate impact on clinical efficacy between HCP and LCP.

It is important to acknowledge some limitations and confounders in our study. First, as a small-scale RCT study the generalizability of our findings is limited. Although disease severity and baseline comorbidities were equally distributed between the study groups, these conditions may potentially confound our observations among the limited number of the recipients. Second, the loss of many patients to follow-up after Day 1 restricted our availability to long-term data, which focuses on the immediate effects of CP. The study inclusion criteria allowed for the recruitment of patients who were not administered systemic corticosteroid at randomization. These criteria resulted in a relatively stable patient population. Asymptomatic VTE screening was not performed. Based on power calculations presented in the Supplemental material of the published trial results, we aimed at recruiting 400 patients to receive either placebo, LCP or HCP. Due to the changing epidemiology and less stringent criteria for dexamethasone treatment in general among hospitalized patients, the number of eligible patients declined and we could recruit only 57 patients for the trial, of which 54 were included in this study, limiting its generalizability.^17^ Additionally, our placebo product was 0.9% normal saline rather than standardized solvent/detergent-treated plasma. This design leaves the impact of plasma products without neutralizing antibodies against the SARS-CoV-2 wild-type virus on acute phase of COVID-19 unexplored.

To summarize, our findings reinforce the previously established significance of platelet-VWF-ADAMTS13 axis in COVID-19.^29^ We have previously shown that different plasma products carry distinct coagulation profiles.^40^ Our novel discovery here suggested that LCP, a modified plasma product, increases VWF:Ag levels and enhances thrombin generation, despite adequate thromboprophylaxis. This underscores the importance of administering only HCP to COVID-19 patients in the acute phase, supporting previous guidance.^27^ Based on our results, HCP administration is associated with less hypercoagulability than LCP, and potentially explains the variability in the efficacy of CP products reported earlier.

## Supplemental Material

sj-docx-1-cat-10.1177_10760296251317522 - Supplemental material for Coagulation Profile of Convalescent Plasma Donors and Recipients Supplemental material, sj-docx-1-cat-10.1177_10760296251317522 for Coagulation Profile of Convalescent Plasma Donors and Recipients by Hanna H Pitkänen, Tuukka Helin, Tamim Khawaja, Jukka-Pekka Pietilä, Mikael Kajova, Hanna Välimaa, Tero Vahlberg, Jarkko Ihalainen, Antti Vierikko, Olli Vapalahti, Anu Kantele and Riitta Lassila in Clinical and Applied Thrombosis/Hemostasis
