# Supplementary material for: Coagulation Profile of Convalescent Plasma Donors and Recipients
Source: Clin Appl Thromb Hemost. 2025 Jan 31;31:10760296251317522. doi: 10.1177/10760296251317522 (PMC11783493; doi:10.1177/10760296251317522)

**Supplemental Figure 1. C-reactive protein (A) and thrombin generation variables (B-D) in standard dose low molecular weight heparin (LMWH) and intermediate dose LMWH groups.**

The recipients who were administered an intermediate dose (I) LMWH had higher CRP on Day 1 than those with standard dose (S) LMWH (A). In the Calibrated Automated Thrombogram, the baseline peak thrombin generation was enhanced in the S LMWH group compared to the I group (B). Both Day 1 lag time (C) and time to peak thrombin (D) were prolonged in the I LMWH dose group. n= the number of recipients in each group. **p=0.026 *p=0.032 ****p=0.002 ***p=0.004


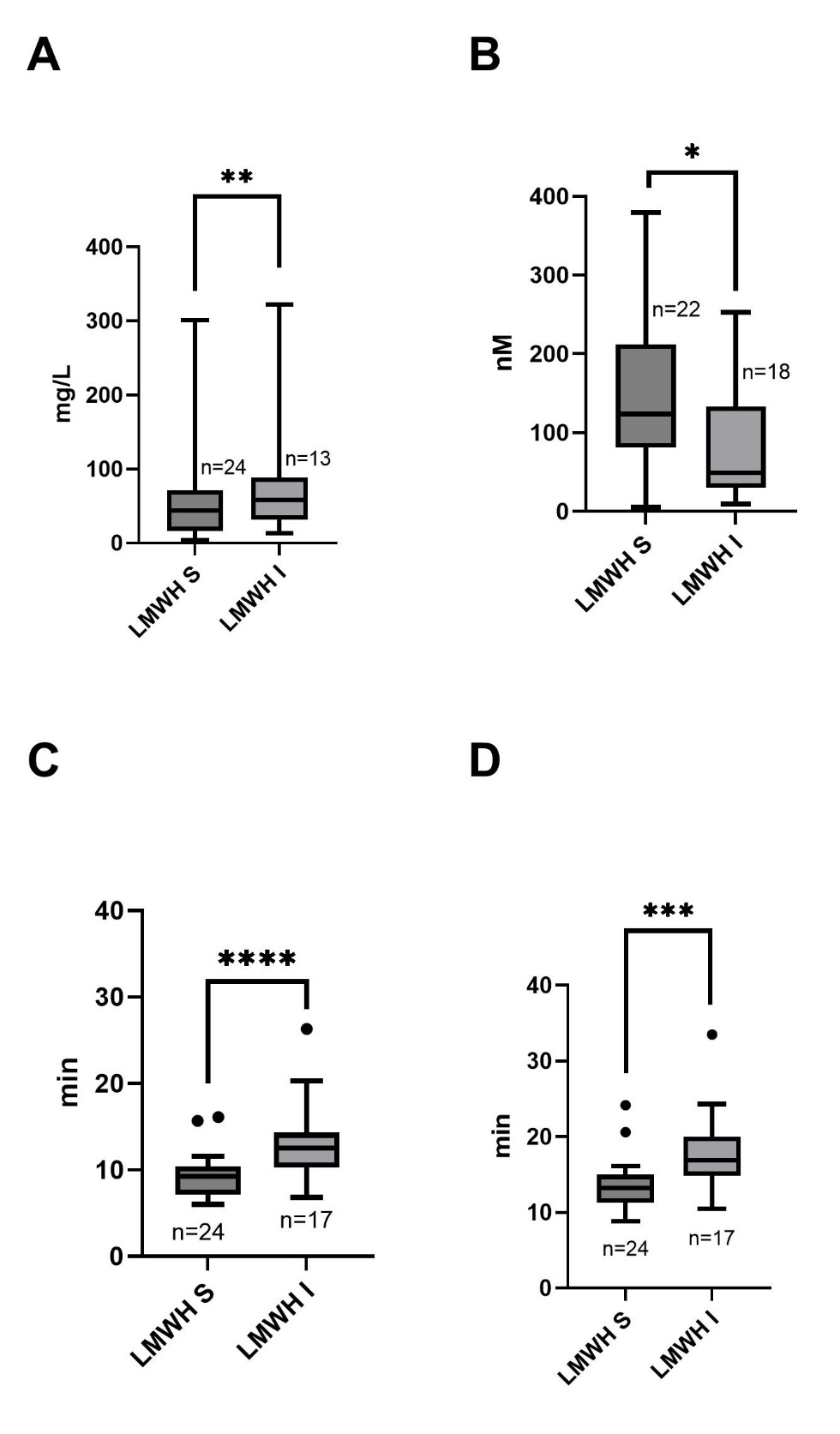

Supplement: sj-docx-1-cat-10.1177_10760296251317522 - Supplemental material for Coagulation Profile of Convalescent Plasma Donors and Recipients [file sj-docx-1-cat-10.1177_10760296251317522.docx]
